# Camphor a Preservative of Ergot of Rye

**Published:** 1845-02

**Authors:** John N. Simpson


*Camphor a Preservative of Ergot of Rye.* By JOHN N. SIMPSON, M. R. C. S., &c.—I was not a little surprised to read some remarks by Mr. Rawle, stating camphor to be a preservative of ergot of rye. I can only say that I have been in the habit of using it for the last nine or ten years, but not exactly in the manner described by him. I order the camphor to be *mixed* with the powdered ergot, in the proportion of a grain in every scruple. By this means I think the camphor is more intimately diffused throughout the whole than can possibly take place by the plan proposed by Mr. Rawle. I do not give this either as a new, or, indeed, my own discovery; for I adopted the method by having seen it in the practice of Mr. Spurgin, an old practitioner also at Saffron Walden, and from whom I have every reason to believe that your correspondent also obtained the same information, he having been in the same gentleman's practice.—*London Lancet.*

